# Correspondence: “Association of CD40 gene polymorphisms and immune thrombocytopenic purpura in the adult Egyptian population”

**DOI:** 10.1007/s44313-025-00066-6

**Published:** 2025-03-07

**Authors:** Elsayed Abdelkreem

**Affiliations:** Department of Pediatrics, Faculty of Medicine, Sohag University, Sohag, 82524 Egypt

To the editor:

We read the paper titled “Association of CD40 gene polymorphisms and immune thrombocytopenic purpura in the adult Egyptian population” published by Ellithy et al. [1], and we would like to explain some of the methodological information provided by the authors, which may impact the interpretation of the reported results.

The authors genotyped the *CD40* rs1883832 (NM_001250.6:c.−1 T > C) polymorphism in the participants using polymerase chain reaction–restriction fragment length polymorphism (PCR–RFLP) with N*coI* restriction endonuclease. This enzyme identifies the CCATGG sequence and cleaves the two cytosines. The presence of the *CD40* rs1883832 T allele alters the sequence to CTATGG, preventing the N*coI* enzyme from performing this cut.

The genotyping criteria for *CD40* rs1883832 appear to be incorrect according to the methodological information provided by Ellithy et al. [1]. The N*coI* cut of the amplified PCR products was reported for the *CD40* rs1883832 T allele in the gel electrophoresis pattern. The presence of two electrophoretic bands of 130 bp and 373 bp was described for the TT genotype, whereas the presence of a single 503 bp band was described for the wild-type (CC) genotype. As such, the genotyping findings and allele frequencies of *CD40* rs1883832 (−1 T > C) reported by Ellithy et al. [1] appear to be incorrect. Accordingly, the analysis and interpretation of ITP predisposition and the response to treatment in association with the *CD40* rs1883832 (−1 T > C) genotype were affected.

Other researchers have genotyped *CD40* rs1883832 (−1 T > C) using the PCR–RFLP technique and have consistently demonstrated that the N*coI* enzyme digests the amplified DNA fragments containing the C allele. These results were validated via direct sequencing [2–5].

Figure 1 in Ellithy et al. [1] contains an error: the change in the *CD40* rs1883832 nucleotide was incorrectly labeled as G > A.

We think that the authors should carefully review their genotyping methods and validate their results considering the above remarks.

## Data Availability

No datasets were generated or analysed during the current study.
